# Rare metachronous isolated splenic metastasis of sigmoid carcinoma: a case report and literature review

**DOI:** 10.1093/jscr/rjaf1098

**Published:** 2026-04-30

**Authors:** Yichao Yan, Xinchun Wu, Junxi Zhu, Yankai Zhang, Lin Chen, Ning Ning

**Affiliations:** Department of Gastrointestinal Surgery, Peking University International Hospital, No.1 Life Park Road, Zhongguancun Life Science Park, Changping District, Beijing, People's Republic of China; Department of Gastrointestinal Surgery, Peking University People's Hospital, No.11 Xizhimen South Street, Xicheng District, Beijing, People's Republic of China; Department of Gastrointestinal Surgery, Peking University People's Hospital, No.11 Xizhimen South Street, Xicheng District, Beijing, People's Republic of China; Department of Gastrointestinal Surgery, Peking University International Hospital, No.1 Life Park Road, Zhongguancun Life Science Park, Changping District, Beijing, People's Republic of China; Department of Gastrointestinal Surgery, Peking University International Hospital, No.1 Life Park Road, Zhongguancun Life Science Park, Changping District, Beijing, People's Republic of China; Department of Gastrointestinal Surgery, Peking University International Hospital, No.1 Life Park Road, Zhongguancun Life Science Park, Changping District, Beijing, People's Republic of China

**Keywords:** isolated splenic metastasis, colorectal cancer, splenectomy, adjuvant chemotherapy, literature review

## Abstract

Splenic metastases are uncommon in solid malignancies, and isolated splenic metastases are even more rare. Current research believes that both anatomical and immunological factors contribute to the rarity of splenic metastasis. We reported a rare case of metachronous isolated splenic metastasis of sigmoid carcinoma in a 58-year-old male. The combination of splenectomy, chemotherapy, and targeted therapy plus anti-PD-1 antibody helped the patient achieve tumor-bearing survival for 32 months. This study reviewed cases of isolated splenic metastasis from colorectal cancer, emphasizing the importance of comprehensive treatment in the treatment of solitary splenic metastasis to reduce the risk of development of new metastasis.

## Introduction

Colorectal cancer (CRC) is the third most common cancer and the second leading cause of cancer-related mortality worldwide [1]. Metastasis is one of the most critical factors affecting patient prognosis [2, 3]. In all CRC patients, 25%–30% will be found to have synchronous metastasis at the time of diagnosis, and 15%–20% will develop metachronous metastasis [4]. The most common metastatic site of CRC is the liver, followed by the lungs [5]. However, the splenic metastasis is much less common; isolated splenic metastases from CRC are extremely uncommon, with fewer than 50 cases documented in English-language literature since its first report in 1969 [6]. Herein, we described a rare case of metachronous isolated splenic metastasis followed by hepatic metastasis in a patient with sigmoid colon cancer. Meanwhile, we reviewed the relevant literature in order to raise awareness of this disease and provide guidance for clinical practice.

## Case report

**Figure 1:**
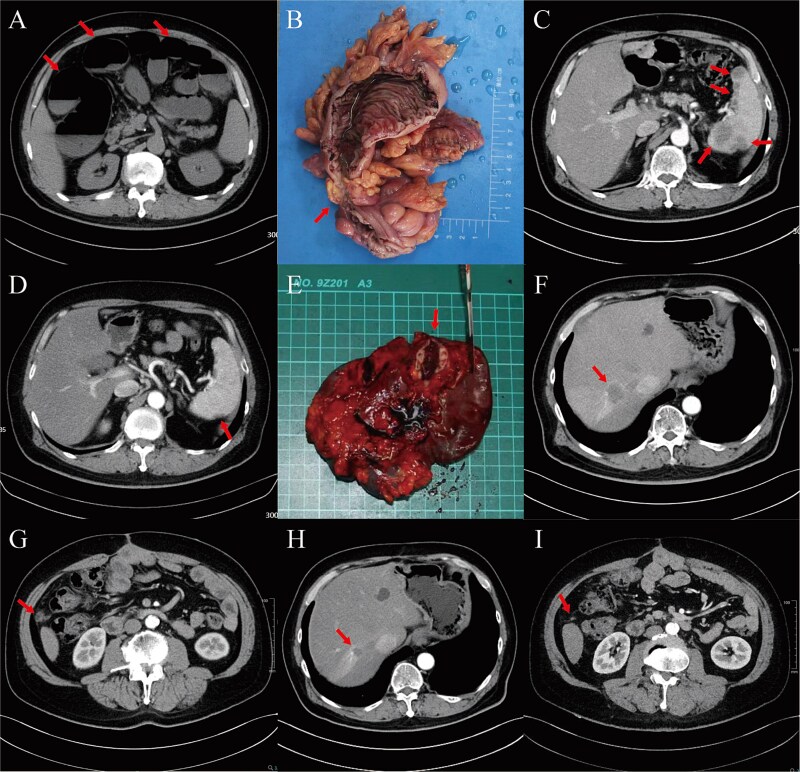
Imaging and operation information of the patient. (A) Emergency CT scan in February 2022. The patient was admitted to our hospital for abdominal pain and distension. CT showed extensive dilatation of the intestine and gas and effusion within the intestine (arrow). (B) Postoperative specimens of emergency sigmoid colectomy revealed a circumferentially growing mass in the sigmoid colon (arrow). (C) Contrast-enhanced CT in February 2023. CT showed multiple metastases in spleen (arrow) but no metastasis elsewhere. (D) Contrast-enhanced CT in August 2023. CT indicated a significant reduction in size and number of splenic metastatic lesions (arrow), with a chemotherapy response evaluated as PR. (E) Postoperative specimens of splenectomy showed splenic metastatic lesions (arrow). (F-G) contrast-enhanced CT in June 2024. CT revealed multiple hepatic (F, arrow) and intra-abdominal metastasis (G, arrow). (H-I) contrast-enhanced CT in October 2024. CT showed significant shrinkage of the intrahepatic metastatic lesion (H, arrow), and the metastatic nodules around the hepatic flexure of colon were not shown (I, arrow).

A 58-year-old male presented with abdominal pain and distention for three days. Emergency evaluation indicated acute complete bowel obstruction (Fig. 1A). Exploratory laparotomy revealed a sigmoid colon mass with dilated bowel loops, and sigmoid colectomy was performed (Fig. 1B). Postoperative pathology confirmed a moderately differentiated tubular adenocarcinoma of the sigmoid colon (5.5 × 5 × 1.4 cm), staged as pT4aN1aM0. Immunohistochemical results: CDX-2(+), Villin(+), Her-2(0), MSH6(+), MSH2(+), MLH1(+), PMS2(+), Ki-67(40%+), CD34(tumor emboli+), D2–40(tumor emboli+), S-100(−), CK(+), CK7(−), CK20(+), SATB2(+). Genetic testing showed mutations in the APC, KRAS, and TP53 genes, with a tumor mutational burden of 1.41 muts/Mb. One month postoperatively, the patient started six cycles of Xelox + Bevacizumab(BEV) chemotherapy, which were well-tolerated. No signs of recurrence or metastasis were noted during this period. Ten months postoperatively, routine follow-up showed CA19–9 and CEA levels were elevated, along with the positive Minimal Residual Disease (MRD). Contrast-enhanced computed tomography (CT) revealed multiple splenic metastases (Fig. 1C). Following multidisciplinary team discussion, the patient was diagnosed with metachronous isolated splenic metastasis of sigmoid colon cancer. Subsequently, the patient received eight cycles of FOLFIRI + BEV + PD-1 inhibitor therapy and achieved partial response (PR) (Fig. 1D). Blood test showed MRD turned negative. After MDT discussion, laparoscopic splenectomy was performed (Fig. 1E). Pathology revealed multifocal subcapsular tubular adenocarcinoma metastases. Immunohistochemistry showed SATB2 (partial+), CDX-2(+), Villin(+), supporting the diagnosis of CRC metastasis. After the operation, the patient was advised to continue combination chemotherapy, targeted therapy, and immunotherapy, but he declined. Nine months later, tumor markers rebounded, enhanced CT revealed multiple hepatic and intra-abdominal metastases (Fig. 1F-G). The patient resumed treatment with eight cycles of FOLFIRI + BEV + PD-1 inhibitor therapy, chemotherapy assessment showed PR again (Fig. 1H-I). Maintenance therapy of capecitabine + BEV + PD-1 inhibitor was initiated and continues. The patient has remained clinically stable and has achieved a survival with tumor of ~32 months at the time of manuscript preparation.

## Discussion

Metachronous isolated splenic metastases are extremely rare [7]. In a 20-year retrospective autopsy study conducted by Schön *et al.*, only 57 cases of splenic metastases (3.0%) were identified among 1898 confirmed malignancy cases [8]. Currently, researchers attribute the rarity of splenic metastasis in solid tumors to three primary factors. First, the tortuous nature of splenic arterial vessels hinders the direct entry of hematogenous tumor cells into the spleen. Second, as an immune organ, the spleen possesses specialized functions and immune microenvironment that are unfavorable for the retention and proliferation of tumor cells. Lastly, while lymphatic metastasis is a critical pathway for the spread of solid tumors, the spleen lacks afferent lymphatic vessels, further limiting the likelihood of metastatic involvement [7]. In our case report, the patient exhibited positive MRD in peripheral blood at the time of splenic metastasis, supporting the notion that hematogenous spread may be a significant pathway for metachronous splenic metastasis in CRC [9]. Notably, we found most patients undergoing splenectomy demonstrated no lymph node metastasis surrounding the spleen. Future research is needed to investigate the role of the splenic immune microenvironment in preventing tumor metastasis, which may provide novel insights into the mechanisms of tumor spread and potential therapeutic strategies.

Surgery remains the primary treatment modality for splenic tumors, with radical splenectomy serving both diagnostic and therapeutic purposes [10]. Based on our literature review, most patients with metachronous isolated splenic metastasis from CRC underwent splenectomy, demonstrating excellent prognostic outcomes. Only five cases developed new metastasis or died postoperatively. In a case reported by Abdou *et al.*, early metastases to the abdominal cavity, mediastinum, and left inguinal lymph nodes occurred 6 months after splenectomy, which the authors attributed to the absence of adjuvant therapy [11]. The patient achieved PR following six cycles of chemotherapy. As for our patient, although the MRD test of peripheral blood was positive, comprehensive evaluation revealed no evidence of distant metastases to other organs. Following adjuvant therapy, the metastatic lesions demonstrated a significant reduction, with MRD turning negative. Consequently, splenectomy was performed as the definitive therapeutic intervention. However, our patient, who declined adjuvant chemotherapy after splenectomy, developed metachronous multiple hepatic metastases 9 months after splenectomy, which was similar to the case reported by Abdou *et al.* These findings align with clinical observations suggesting that adjuvant chemotherapy plays a crucial role in reducing new metastasis risk. Based on our analysis and literature review, we recommend adjuvant chemotherapy for patients with isolated splenic metastases from CRC, particularly for those with known genetic mutations or MRD positivity prior to surgery, to improve long-term outcomes and minimize metastasis or recurrence.

In conclusion, we reported a rare case of metachronous isolated splenic metastasis with hepatic metastases in a patient with sigmoid colon cancer. Based on our literature review, radical splenectomy should be regarded as the primary treatment of isolated splenic metastasis, with favorable outcomes observed in most cases. Besides, this case underscores the critical role of adjuvant chemotherapy, particularly in patients with MRD positivity or genetic mutations, to reduce new metastases risk.
